# Microglia Express Mu Opioid Receptor: Insights From Transcriptomics and Fluorescent Reporter Mice

**DOI:** 10.3389/fpsyt.2018.00726

**Published:** 2019-01-04

**Authors:** Tando Maduna, Emilie Audouard, Doulaye Dembélé, Nejma Mouzaoui, David Reiss, Dominique Massotte, Claire Gaveriaux-Ruff

**Affiliations:** ^1^Institut de Génétique et de Biologie Moléculaire et Cellulaire, Illkirch, France; ^2^Université de Strasbourg, Illkirch, France; ^3^Centre National de la Recherche Scientifique, Illkirch, France; ^4^Institut National de la Santé et de la Recherche Médicale, Illkirch, France; ^5^Ecole Supérieure de Biotechnologie de Strasbourg, Illkirch, France; ^6^CNRS UPR3212, Institut des Neurosciences Cellulaires et Intégratives, Centre National de la Recherche Scientifique, Université de Strasbourg, Strasbourg, France

**Keywords:** microglia, opioid receptor, mu, transcriptomics, gene clusters, fluorescent reporter mice, analgesic tolerance, opioid-induced hyperalgesia

## Abstract

**Background:** Microglia activation contributes to chronic pain and to the adverse effects of opiate use such as analgesic tolerance and opioid-induced hyperalgesia. Both mu opioid receptor (MOR) encoded by *Oprm1/OPRM1* gene and toll like receptor 4 (TLR4) have been reported to mediate these morphine effects and a current question is whether microglia express the Oprm1 transcript and MOR protein. The aim of this study was to characterize *Oprm1*-MOR expression in naive murine and human microglia, combining transcriptomics datasets previously published by other groups with our own imaging study using the Cx3cr1-eGFP-MOR-mCherry reporter mouse line.

**Methods:** We analyzed microglial *Oprm1/OPRM1* expression obtained from transcriptomics datasets, focusing on *ex vivo* studies from adult wild-type animals and adult post-mortem human cerebral cortex. *Oprm1*, as well as co-regulated gene sets were examined. The expression of MOR in microglia was also investigated using our novel fluorescent Cx3cr1-eGFP-MOR-mcherry reporter mouse line. We determined whether CX3cR1-eGFP positive microglial cells expressed MOR-mCherry protein by imaging various brain areas including the Frontal Cortex, Nucleus Accumbens, Ventral Tegmental Area, Central Amygdala, and Periaqueductal Gray matter, as well as spinal cord.

**Results:** *Oprm1* expression was found in all 12 microglia datasets from mouse whole brain, in 7 out of 8 from cerebral cortex, 3 out of 4 from hippocampus, 1 out of 1 from striatum, and 4 out of 5 from mouse or rat spinal cord. *OPRM1* was expressed in 16 out of 17 microglia transcriptomes from human cerebral cortex. In Cx3cr1-eGFP-MOR-mCherry mice, the percentage of MOR-positive microglial cells ranged between 35.4 and 51.6% in the different brain areas, and between 36.8 and 42.4% in the spinal cord.

**Conclusion:** The comparative analysis of the microglia transcriptomes indicates that *Oprm1/OPRM1* transcripts are expressed in microglia. The investigation of Cx3cr1-eGFP-MOR-mCherry mice also shows microglial expression of MOR proteinin the brain and spine. These results corroborate functional studies showing the actions of MOR agonists on microglia and suppression of these effects by MOR-selective antagonists or MOR knockdown.

## Introduction

Activation of the mu opioid receptor (MOR), encoded by the Oprm1/OPRM1 gene in rodents and humans, respectively (1, 2), mediates opioid analgesia and the adverse consequences of opioid use (3, 4). Glial cells and in particular microglia are known to contribute to chronic pain (5) as well as to opioid tolerance and opioid-induced hyperalgesia (OIH) (6–8). However, whether microglia express *Oprm1* and whether microglial *Oprm1* would have a role in chronic pain and other opioid effects remains to be demonstrated. Most studies reporting MOR expression or function in microglia have been performed on cultured microglia (9–18). It has been shown however that gene expression profiles differ between microglia in culture and adult mouse microglia *in vivo* (19) and therefore the demonstration of MOR expression in cultured microglia does not allow to conclude for MOR expression in adult microglia *in vivo*. Horvath et al. (20) have shown MOR expression in rat spinal microglia *in vivo* by immunohistochemistry. However, two other studies have contradicted these findings by showing a lack of MOR expression in spinal cord microglia (21, 22). Thus, whether microglia in adults express MOR still remains an unsolved question. Specifically, Corder et al. present considerable evidence that argues against the expression of MOR messenger and protein in mouse spinal cord microglia. Their findings are further strengthened by transcriptomic analyses which show a lack in co-expression of Oprm1 mRNA with microglial markers (21). However, critical analysis and commentary on these interesting results is not possible yet due to the limited access of the datasets used for transcriptomic analysis. Therefore, whether *Oprm1/OPRM1* are expressed by microglia remains a matter of debate that should be further investigated. To date, there are no published studies focusing on the analysis of a large series of transcriptomic datasets for Oprm1 expression in microglia that would allow assessing, unambiguously, *Oprm1* expression in microglia *in vivo*. In addition, *OPRM1*/MOR expression in human microglia is yet to be fully characterized. For this purpose, we have used novel approaches to characterize *Oprm1/OPRM1* expression in microglia based on transcriptomics and have used fluorescent reporter mice to characterize MOR expression *in vivo*.

A number of laboratories have generated transcriptomics datasets for microglia that can be used for analyzing gene expression profiles (23, 24) as well as for investigating microglia physiology and their responses in disease (25, 26). We have analyzed published datasets from microarray (MA) and RNA-sequencing (RNA-seq) studies performed on rodent and human microglia for Oprm1 and OPRM1 gene expression. We have completed this analysis by imaging microglial MOR using a novel double fluorescent Cx3cr1-eGFP-MOR-mCherry mouse line. The chemokine receptor CX3CR1 is a specific marker for phagocytic cells and labels specifically microglia and macrophages in the nervous system of naive animals (27–29). The Cx3cr1-eGFP mouse line was originally used to explore CX3CR1 function (30) then later to map the fate of tissue macrophages including microglia (28, 29). A reporter knock-in mouse line for MOR, the MOR-mCherry line, allows to map the distribution of MOR-expressing cells in mice using fluorescence imaging (31). In order to localize MOR protein in microglia, we have bred these two lines together to generate the Cx3cr1-eGFP-MOR-mCherry mouse line. Thus, we have analyzed MOR expression in various brain regions implicated in chronic pain or chronic opiate effects as well as the spinal cord in control non-pathological conditions. In addition, as sex differences are an important factor for chronic pain (32–34) and greatly impact the microglial contribution to pain (35, 36), we investigated Oprm1/OPRM1-MOR presence in microglia from both females and males.

## Materials and Methods

### Transcriptomics Analyses

As microglia develop after birth until post-natal day 15, followed by stabilization of microglial numbers (37–39), we focused the Oprm1 expression analysis on microglia from juvenile-adult wild-type naïve rodent and juvenile-adult humans with no reported pain phenotype. The datasets used and related information including the pathology of patients from whom microglia were collected, are indicated in Supplementary Tables 1–4. The selection criteria for which datasets to include in the present study were as follows: normalized mouse datasets included in the database recently published by Friedman et al. (40) (see Data S2 in Friedman et al.) and additional normalized datasets containing *Oprm1* in their gene list; from wild-type mice aged 0.5 months and older. Microglia datasets from mouse whole brain were from the studies by Wang et al. (41), Verheijden et al. (42), Poliani et al. (43), Erny et al. (44), Szulzewsky et al. (45), Pyonteck et al. (46), Bruttger et al. (47), Lavin et al. (48), Bennett et al. (49), Gosselin et al. (50), Krasemann et al. (51), and Zhao et al. (52). Microglia datasets from mouse brain areas were from Orre et al. (53), Arumugan et al. (54), Grabert et al. (55), Friedman et al. (40), Srinivasan et al. (56), Zhang et al. 2014 (57), and Matcovitch-Natan et al. (58). Microglia datasets from rodent spinal cord were from Chiu et al. (59), Denk et al. (60), Noristani et al. (61), Matcovitch-Natan et al. (58), and Jokinen et al. (62). Information on these datasets including publication authors and year, accession number, mouse or rat strain, sex, age, dissociation, and microglia isolation methods, and genomics assay are given in Supplementary Tables 1–4.

Human datasets comprised those published by Zhang et al. (63) and contained in Friedman's Supplementary Table 2 as well as normalized datasets recently reported by Gosselin et al. (64) and Galatro et al. (65) as described in Supplementary Table 4. Normal cortical regions were resected from patients with diseases described in column F. Among the 19 datasets by Gosselin et al. we selected 10 datasets derived from individuals aged 13 and older. Among the 39 datasets from adult individuals by Galatro et al. we selected 8 datasets derived from samples collected at a maximum delay of 10 h post-mortem. Information on these datasets including publication authors and year, accession number, sex, age, cortex area, dissociation, and microglia isolation methods, and genomics assay are given in Supplementary Table 4.

In addition to our main focus set, (A) showing only the expression levels of *Oprm1* gene transcript from purified microglia, three other groups of gene clusters were formed: (B) Myeloid cells, (C) Activation patterns and (D) Neurons and Astrocytes. The list of genes contained in each of the gene clusters of B, C, and D was formed using the gene lists defined in Friedman et al. (40) and filtered to exclude the few genes expressed by any other cell type. The Myeloid gene clusters (B) include the Microglia, the Macrophage, and the Neutrophil-Monocytes gene clusters, respectively. The activation pattern clusters (C) comprise the Interferon-related, Proliferation-related, LPS-related and Neurodegeneration-related gene clusters. The Neurons and Astrocyte gene clusters (D) are composed of the Neuron-associated, Excitatory neuron-associated, GABAergic neuron-associated, and Astrocyte-associated genes clusters.

**Figure 1:**
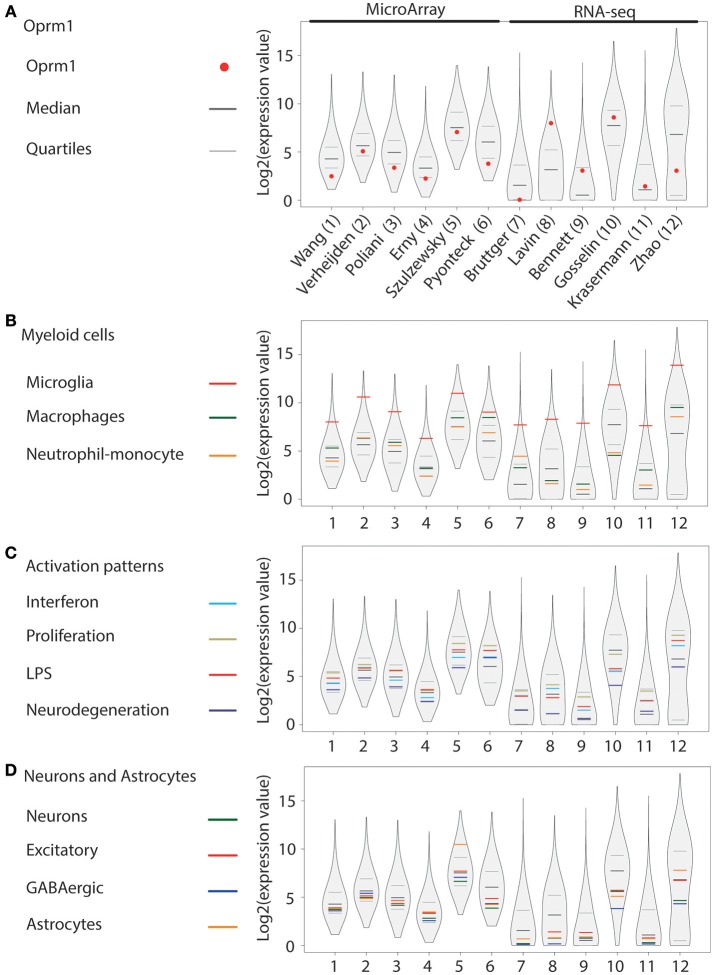
*Oprm1* and gene clusters expression in mouse whole brain microglia datasets. Expression levels of the genes identified in each dataset are represented in violin plots demonstrating the median, as well as the 25th and 75th quartiles. Datasets are represented by name of the first author in **(A)**, which are denoted numerically in **(B–D)**. **(A)** Expression of *Oprm1* is below the median in purified microglia assayed with MA, and fluctuates above and below the median in purified microglia assayed with RNA-seq. **(B)** Expression values of the Myeloid cell clusters demonstrate a high expression of Microglia-related genes which are in the 75th quartile in all the datasets analyzed. **(C)** Expression values of the Activation pattern clusters are below the 75th quartile in all but one dataset (dataset #6). **(D)** Expression values of the Neurons and Astrocytes clusters are below the median in 5 out 6 of the datasets derived from MA assays, whereby the Astrocyte-related genes have expression values above the 75th quartile in dataset #5. When assayed with RNA-seq, the Astrocyte-related genes are below the median, except for dataset #12, where the values are above the median.

**Figure 2:**
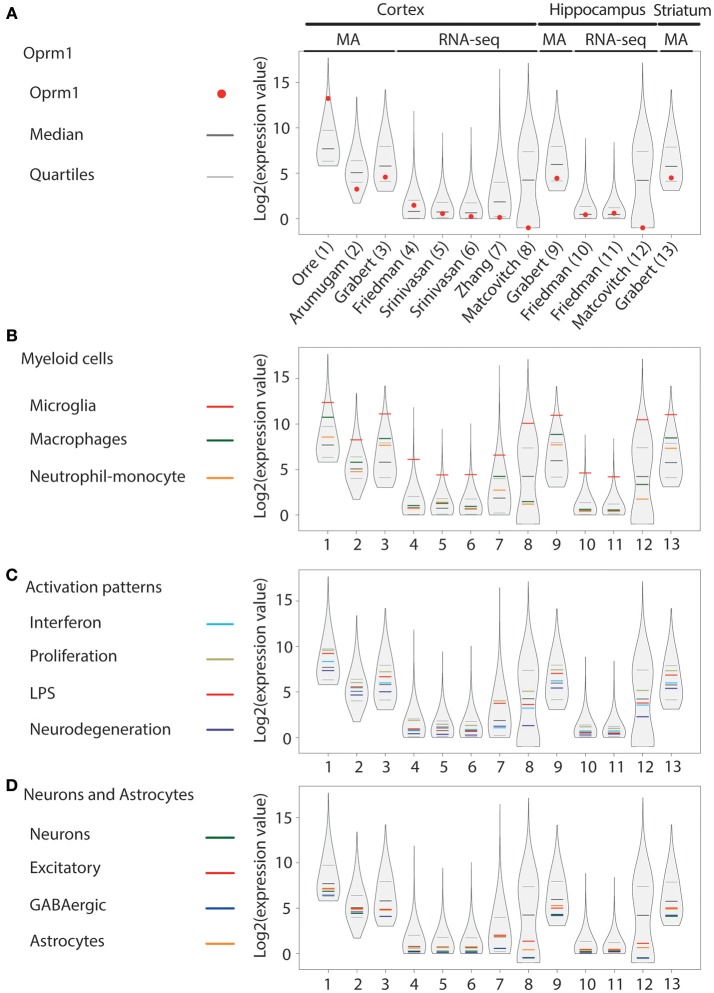
*Oprm1* and gene clusters expression in mouse cortex, hippocampus and striatum microglia datasets. Expression levels of the genes identified in each dataset are represented in violin plots demonstrating the median, as well as the 25th and 75th quartiles. Datasets are represented by name of the first author in **(A)** which are denoted numerically in **(B–D)**. In mouse cortex and hippocampus, microglia were assayed with either microarrays (MA) or RNA-sequencing (RNA-seq) as underlined on **(A)** and assayed with MA only in the striatum (dataset #13). **(A)** Oprm1 expression values were distributed below and above the median independent of the assay used to measure gene expression. RNA-seq datasets (#8 and 12) yielded to no expression of *Oprm1* in microglia. The highest Oprm1 expression value was yielded by dataset #1 in cortical microglia. **(B)** Among the Myeloid gene clusters, the Microglia-related gene module was the mostly highly expressed in all datasets analyzed, with expression values distributed about the 75th quantile. **(C)** Proliferation-related genes were the most highly expressed within the Activation pattern clusters, distributed within the 75th quantile. **(D)** Neuron-, GABAergic-, and Astrocyte-related genes had expression values that were below the median in most of the datasets analyzed. In the cortex and hippocampus, the Excitatory neuron- and Astrocyte-related genes are distributed along the median for datasets #5–7 and #10, 11.

**Figure 3:**
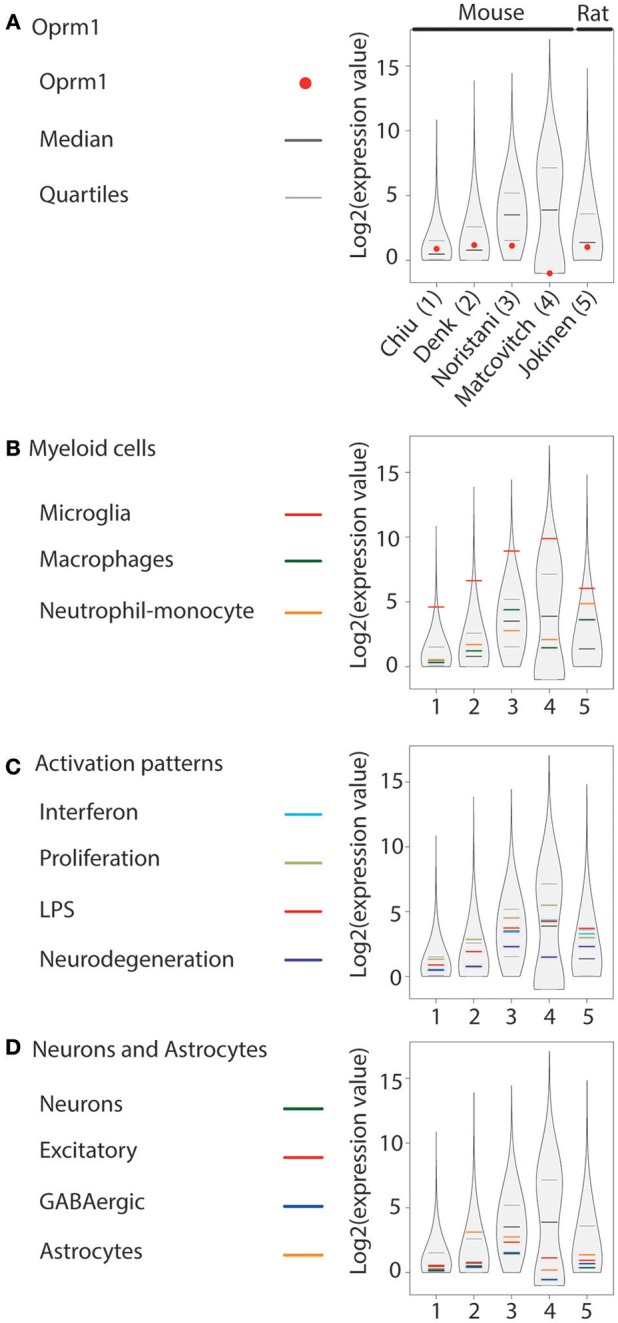
*Oprm1* and gene clusters expression in rodent spinal cord microglia datasets. Expression levels of the genes identified in each dataset are represented in violin plots demonstrating the median, as well as the 25th and 75th quartiles. Datasets are represented by name of the first author in **(A)** which are denoted numerically in **(B–D)**. Gene expression in the spinal cord was assayed with RNA-seq exclusively. **(A)** Oprm1 expression values ranged from above the median to undetected level (#4). **(B)** In the Myeloid gene cluster, the Microglia- related genes were the mostly highly expressed in all datasets analyzed, with expression values distributed about the 75th quantile. **(C)** Proliferation-related genes were the most highly expressed within the Activation pattern clusters, except in dataset#5, which had higher expression values for the LPS module in microglia purified from rat. Overall, the expression values of Activation patterns gene clusters were low compared to the Microglia-related genes. **(D)** Neuron-, GABAergic- and Astrocyte-related genes had expression values that were low compared to the Microglia-related genes except for dataset #2 that show high Astrocyte-related module expression **(B)**.

**Figure 4:**
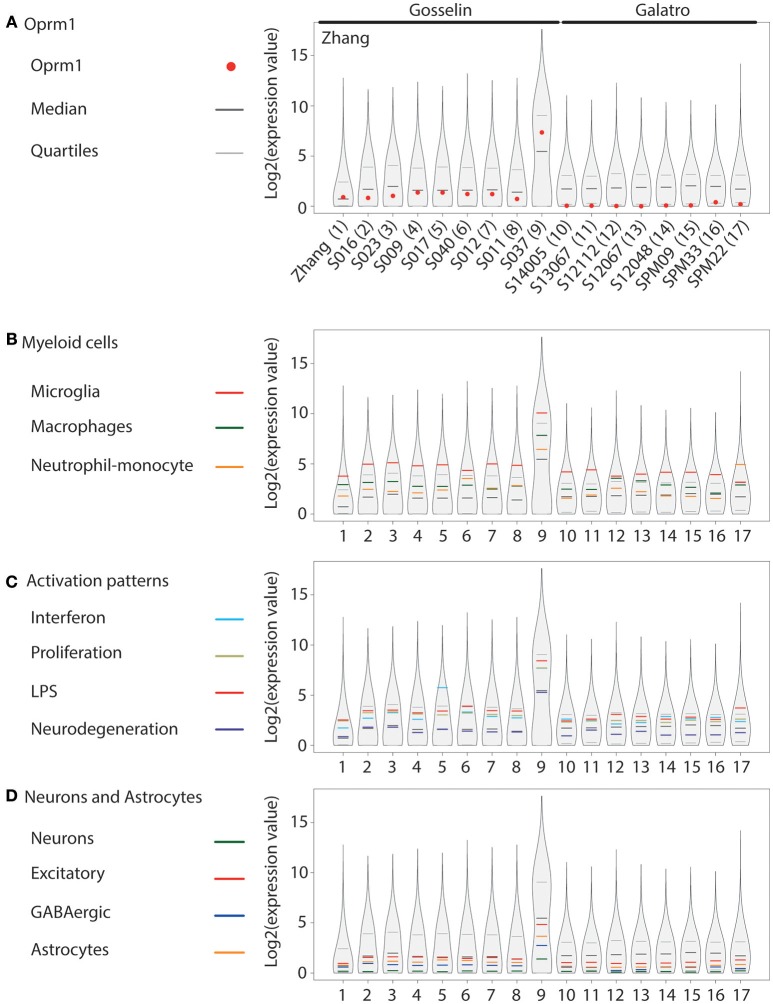
*Oprm1* and gene clusters expression in human cortex microglia datasets. Expression levels of the genes identified in each dataset are represented in violin plots demonstrating the median, as well as the 25th and 75th quartiles. In all datasets, microglia were assayed with RNA-seq. Gosselin datasets (#2–9) comprised of microglia isolated from young males and females, while the other human datasets were from adult and aged postmortem samples of unknown gender (#1) or exclusively males (10–17). Datasets are represented by name of the first author **(A)**, which are denoted numerically in **(B–D)**. **(A)** Oprm1 expression values were distributed below or along the median in the Zhang and Gosselin datasets (#1 and 2–9) but consistently below the median in all the Galatro datasets. **(B)** In the Myeloid gene clusters, the Microglia- related genes were the most highly expressed in all datasets analyzed, with expression values distributed above the 75th quantile, except in dataset #17 which had higher expression values for the Monocytes-Neutrophils-related genes. **(C)** Activation pattern gene clusters were highly expressed in purified human microglia, distributed within, and above the 75^th^ quantile. The majority of the datasets reported the highest expression values for the LPS-related genes. Interferon-related genes had the highest expression values in the datasets #5, 10, 14, and 16 with causes of death associated with blood circulation and aneurysm. **(D)** Neuron-, GABAergic-, and Astrocyte-related genes had expression values along and below the median in dataset#1 and those provided by Gosselin (#2–9). Expression values were all below the median in the aged samples (#10–17).

The logarithm scale two (log2) transformation was applied to each data sample values and 1 was added to expression values to avoid indetermination for zero count reads. For each data sample, the first (25th percentile), the second (median), and the third quantile (75th percentile) values were computed. The log2 values for the Oprm1 transcript were also obtained for each dataset. The log2 values associated with each gene subset of B, C, and D categories were isolated for each sample and an average value was calculated for each subset. The R environment (version 3.5.0) was used to create the basic figures. The vioplot R package was used to obtain a violin plot like contour for the log2 values of each data sample. The log2 values and the 25th, 75th percentile and median values were superimposed on the violin plots using different colors as shown in Figures 1–**4**.

For determining correlations between *Oprm1/OPRM1* expression and the expression of the gene clusters described above, z-scores transformations (1+log2 values) were calculated from the mean expression level of each cluster for each dataset included in the study. The Orre et al. (53) mouse cortex dataset and the S037 dataset of the Gosselin et al. study (64) were removed from the analysis as they varied substantially from all other datasets within their group. The correlation analysis for human datasets included all dataset except for S037 set.

### Cx3cr1-eGFP-MOR-mCherry Mouse Line

#### Animals and Ethics Statement

The animals were housed under standard light, temperature, and humidity conditions (12 h light-dark cycle, 21 ± 1 °C, 55 ± 10% humidity) with food and water *ad libitum*. Brains and spinal cords were collected from male and female mice aged between 5 and 15 weeks. All experiments were conducted respecting the European Communities Council Directives of 22 September 2010 (directive 2010/63/UE) under the guidelines of the Committee for Research and Ethical issues of IASP published in PAIN, 1983; 16:109-110, and were approved by the local ethical committee (Com'Eth d'Ethique pour l'Expérimentation Animale IGBMC-ICS, license N°17) with the agreement number 00876-02.

#### Cx3cr1-eGFP-MOR-mCherry Mice

The Cx3cr1-eGFP-MOR-mCherry mouse line was generated by crossing Cx3Cr1-eGFP mice (29) and MOR-mCherry mice (31) to obtain viable heterozygous animals. These were intercrossed to generate homozygous Cx3Cr1-eGFP-MOR-mCherry mice that are fertile and develop normally. Genotyping was performed by PCR to detect both Cx3Cr1-GFP sequence (35 cycles at 94°C for 30 s, 65.5°C for 30 s, and 72°C for 2 min) using the following primer sequences: Cx3Cr1-Fwd: 5′-TTCACGTTCGGTCTGGTGGGAAATC-3′, Cx3Cr1- Rev:

5′-TTCCTAGTGGAGCTAGGGTCGGGG-3′, eGFP-Fwd:

5′-GATCACTCTCGGCATGGACG-3′, and MOR-mCherry sequence (35 cycles at 94°C for 1 min, 63°C for 1 min, and 72°C for 1 min) using a forward primer located on exon four of Oprm1 gene, -Fwd: 5′-TGACGTGACATGCAGTTGAGATTT-3′and a reverse primer located in the 3' UTR region, Rev: 5′-TCCCACAAACCCTGACAGCAAC-3′. Both female and male Cx3cr1-eGFP-MOR-mCherry mice were analyzed for MOR expression in microglia (3 animals per sex).

#### Tissue Preparation

Mice were deeply anesthetized with intraperitoneal administration of 100/5 mg/kg ketamine/xylazine (Virbac, Carros, France; Rompun, Bayer, La Garenne Colombes, France) and were intracardially perfused with 4% Paraformaldehyde (PFA) in Phosphate Buffer Saline (PBS) solution (4%PFA/PBS). The whole brain and spinal cord were isolated and post-fixed overnight at 4°C in 4%PFA/PBS. Samples were rinsed three times in PBS and cryoprotected in a sucrose/PBS gradient (10, 20, and 30%) for 24 h each. Tissues were embedded in OCT (Tissue Tek, Sakura Fine Technical, Torrance) and coronally (brain) or transversally (spinal cord) sectioned at 30 μm on Superfrost microscope slides using a cryostat (Leica CM3050S). Cryosections were dried at room temperature and then stored at −20°C before imaging.

#### Immunohistochemistry and Quantifications

For immunohistochemistry, cryosections were rehydrated with PBS and were then incubated in a blocking solution (10% horse serum/0.1% Triton X-100/PBS) for 30 min at room temperature. The sections were incubated with the primary antibodies diluted in 10% normal horse serum/0.1% Triton X-100/PBS overnight at 4°C. The sections were then washed 3 times in 0.1% Triton X-100/PBS and incubated with secondary antibodies and Hoechst for 1 h at room temperature, in the same solution as the primary antibodies. After washing 3 times in 0.1% Triton/PBS and once in PBS alone, slides were mounted with the S3023 aqueous mounting medium (DAKO). For staining cis-Golgi, trans-Glogi, and lysosomes, the rabbit polyclonal anti-GM130 antibody (11308-1-AP, Proteintech), the rabbit polyclonal anti-TGN38 antibody (NBP1-03495, Interchim), and the mouse monoclonal anti-Lamp1 antibody (H4A3, Developmental Studies Hybridoma Bank DSHB) were used. The optimal dilutions were set up in preliminary experiments. Anti-GM130, anti-TGN38, and anti-Lamp1 were used at dilutions 1:500, 1:200, and 1:200 (0.6, 5, 0.1 μg/ml), respectively. The secondary antibodies used were the Cy5 conjugated donkey anti-mouse (1:1,000, 715-176-150, Jackson) and the Cy5 conjugated donkey anti-rabbit (1:500, 711-176-152, Jackson) Ig antibodies. Images were acquired with a confocal microscope (8UV, Leica Microsystem) using a 40x objective. The FIJI package for ImageJ software was used for image analysis (66). The percent of MOR-positive microglia among the CX3CR1-eGFP-positive microglia in the different brain and spinal cord regions were quantified on multiple microscopic fields from 3 female and 3 male adult animals. The number of fields counted and of cells examined are indicated in the Supplementary Table 5.

### Statistical Analysis

Statistical analyses were performed with GraphPad Prism version 6.01 to investigate the correlations between Oprm1 expression and the different clusters mentioned above, as well as to compare MOR expression in CX3CR1-eGFP-positive microglia between male and female mice. *Oprm1* and gene module z-scores were all non-normally distributed and correlations were assessed with the Spearman's correlation test. The mean percentage of MOR-positive and CX3CR1-eGFP-positive microglia were tested for normality as well and compared using the Unpaired Student's *t*-test or Mann Whitney test where applicable. The results are presented as the mean ± SEM for microglia cell counts (percentages). A *p*-value of 0.05 or less was considered to be statistical significant.

## Results

### *Oprm1/OPRM1* Expression in Rodent and Human Microglia Transcriptomics Datasets

#### Microglia From Mouse Whole Brain

*Oprm1* was expressed in the twelve datasets analyzed (Figure 1A). Among those, six were issued from MA, see references (41–46) and six from RNA-seq, see references (47–52), as described in Supplementary Table 1. *Oprm1* levels ranged from above the median (*n* = 4) to between the median and first quartile (*n* = 4) and below the first quartile (*n* = 4). *Oprm1* expression tended to be more homogenous in MA than in RNA-seq datasets. The Myeloid gene modules defined by Friedman et al. (40) included Microglia, Macrophage, and Monocyte-neutrophil gene clusters. The Microglia gene cluster was highly expressed in all datasets, at the highest level of all gene clusters (Figure 1B). Macrophage and Neutrophil-monocyte cluster expression was distributed over and under the median among datasets (Figure 1B). The Proliferation cluster stood out as the most expressed among the Activation pattern gene sets, followed by the LPS, or Interferon and then Neurodegeneration gene sets (Figure 1C). The three neuronal (Neurons, Excitatory and GABAergic) gene sets were positioned below the median in nine of the datasets (Figure 1D). The Astrocyte cluster was below the median in eight of the datasets (Figure 1D).

#### Microglia From Murine Cortex, Hippocampus, and Striatum

In the cortex, *Oprm1* expression was expressed in seven out of the eight datasets generated by either MA (53–55) or RNAseq (40, 56, 57) and not detected in the dataset by Matcovitch-Natan et al. derived from Massively parallel Single-cell RNA-seq (Mars-seq) (58). Expression ranged from above the median in two datasets, between median, and first quartile in three datasets, below the first quartile in two datasets, to not being expressed in the Mars-seq dataset (Figure 2A). The dataset with very high *Oprm1* expression was generated using the Agilent MA technology (53) while Affymetrix arrays were used in the other MA studies. In the hippocampus, *Oprm1* was above the median in one dataset, ranged between the median, and first quartile in two datasets, but not expressed in the Mars-seq dataset. In the striatum dataset by Grabert et al., the *Oprm1* was above the first quartile. The Microglia gene cluster was also highly expressed and the highest in all datasets (Figure 2B), similarly to whole mouse brain datasets. Macrophage and Neutrophil-monocyte module expression ranged among datasets from over to under the median, with the Macrophage cluster higher than that of Neutrophil-monocyte (Figure 2B). The Proliferation cluster stood out as the most expressed among the Activation pattern gene sets, followed by the LPS or Interferon and then Neurodegeneration gene sets (Figure 2C), comparably whole mouse brain data. The neuronal (Neurons, Excitatory and GABAergic) as well as the Astrocyte gene set were positioned below the median in nine of the datasets (Figure 2D).

#### Rodent Spinal Cord

The expression of *Oprm1* was analyzed in four mouse and one rat spinal microglia datasets obtained by RNA-seq (58–62). *Oprm1* is expressed above the median in two mouse spinal cord datasets, below the first quartile in one data set, and not expressed in the Mars-seq dataset by Matcovitch-Natan et al. The rat spinal cord dataset showed *Oprm1* level above the first quartile (Figure 3A). The Microglia gene cluster was also highly expressed in the five datasets (Figure 3B). Macrophage and Neutrophil-monocyte cluster expression varied among datasets from over to below the median (Figure 3B). In the Activation pattern gene modules, the Proliferation gene sets were the most expressed in mouse but not rat, whereas Neurodegeneration gene sets showed the lowest level (Figure 3C), similarly to what was found in mouse brain. The three neuronal (Neurons, Excitatory and GABAergic) gene sets were positioned below or at the median level while Astrocyte cluster expression differed across the datasets (Figure 3D).

#### Human Cortex

*OPRM1* level was analyzed in microglia RNA-seq datasets generated from temporal cortex gray matter (63), different cortical areas (64), and parietal cortex (65) (Figure 4A). *OPRM1* is expressed above the median in the dataset generated by Zhang et al. (63) for which microglia were purified from cortex gray matter collected on juvenile-adult individuals. In the study by Gosselin et al. (64) on cortical microglia enriched from young adults, *OPRM1* levels ranged between the median and first quartile. In the S037 dataset issued from microglia collected from a brain tumor, *OPRM1* was highly expressed. In the microglia datasets by Galatro et al. (65), collected from the parietal cortex of aged people (57–102 years), *OPRM1* was around the first quartile and not expressed in the S12067 sample (Figure 4A). The Microglia gene module was the most expressed in all sets except one and the Macrophage cluster had generally a higher level than the Neutrophil-monocyte cluster (Figure 4B). Among the activation pattern gene sets, the LPS and Interferon sets showed mostly the highest levels, followed by the Proliferation cluster. The Neurodegeneration gene sets showed the lowest level of expression (Figure 4C). The values for the Excitatory and GABAergic clusters were higher than those of the Neurons cluster, which showed the lowest expression levels (Figure 4D), differing from mouse datasets. The Astrocyte cluster was below the median on most datasets (Figure 4D).

#### Single Cell RNAseq Data Sets

We investigated *Oprm1* expression in microglia datasets from published single cell RNA-seq studies. Transcripts for *Oprm1* were not found in the datasets which contained about 1,300, 1,985, 6,000, 1,179, 1,900–3,300, and 1,169 and 800 genes expressed per cell, respectively (27, 67–72). Microglia datasets generated from microdissected basal ganglia nuclei led to the identification of 2,647 expressed genes per cell that did not include *Oprm1* (73).

### Relationships Between Oprm1 Expression and Gene Clusters

#### Myeloid Cells

**Figure 5:**
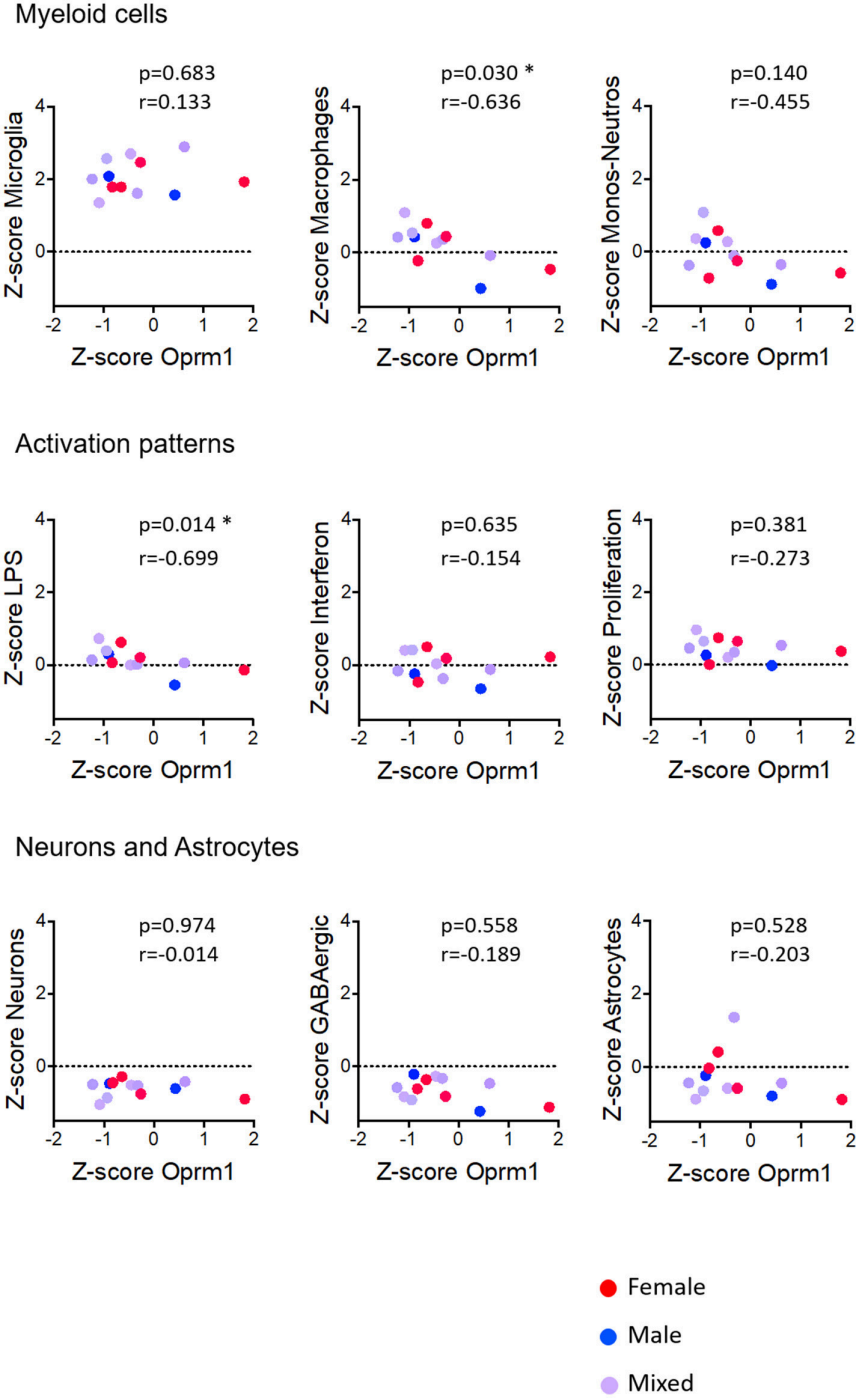
Correlation of Oprm1 gene expression within the Myeloid cells, Activation patterns, and Neurons and Astrocytes gene clusters in mouse whole brain datasets. Oprm1 expression does not correlate with the microglia-related and the Monocyte-Neutrophil-related genes (*p* >0.05, ns) but is significantly negatively correlated with the Macrophages-related genes (*r* = 0.636; *p = 0.03). Oprm1 expression has a negative correlation (*r* < 0) with all the Activation patterns-, Neurons, and Astrocytes gene clusters, which is only statistically significant for the LPS-related gene module (*p = 0.014). All correlations were determined with the Spearman's correlation test.

**Figure 6:**
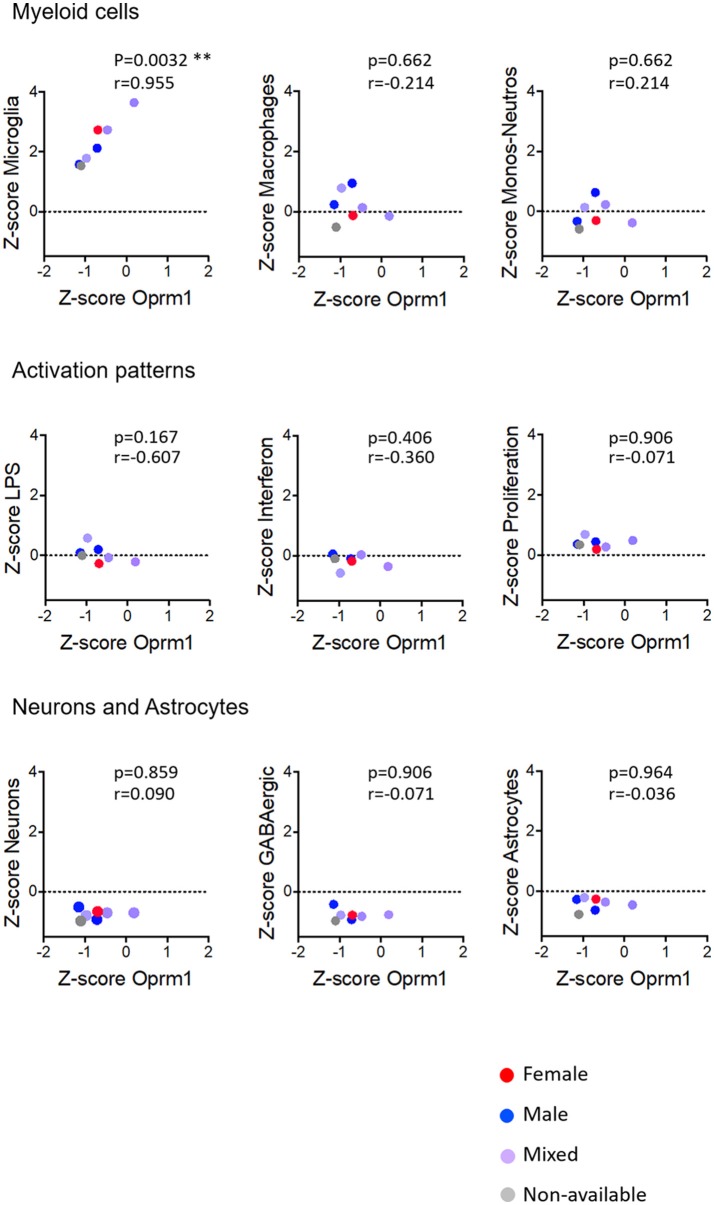
Correlation of Oprm1 gene expression within the Myeloid cells, Activation patterns, and Neurons and Astrocytes gene clusters in mouse cortex datasets. Oprm1 expression is positively and significantly correlated with the microglia-related genes (*r* = 0.955; **p = 0.0032) but does not correlate with the Macrophages- and Monocyte-Neutrophils-related genes (*r* ≤ 0.214*; p* > 0.05, ns). Oprm1 expression does not correlate with any of the Activation patterns- (*r* < 0*; p* > 0.05, ns), Neurons or Astrocytes gene clusters (*r* ≤ 0.09*; p* > 0.05, ns). All correlations were determined with the Spearman's correlation test.

**Figure 7:**
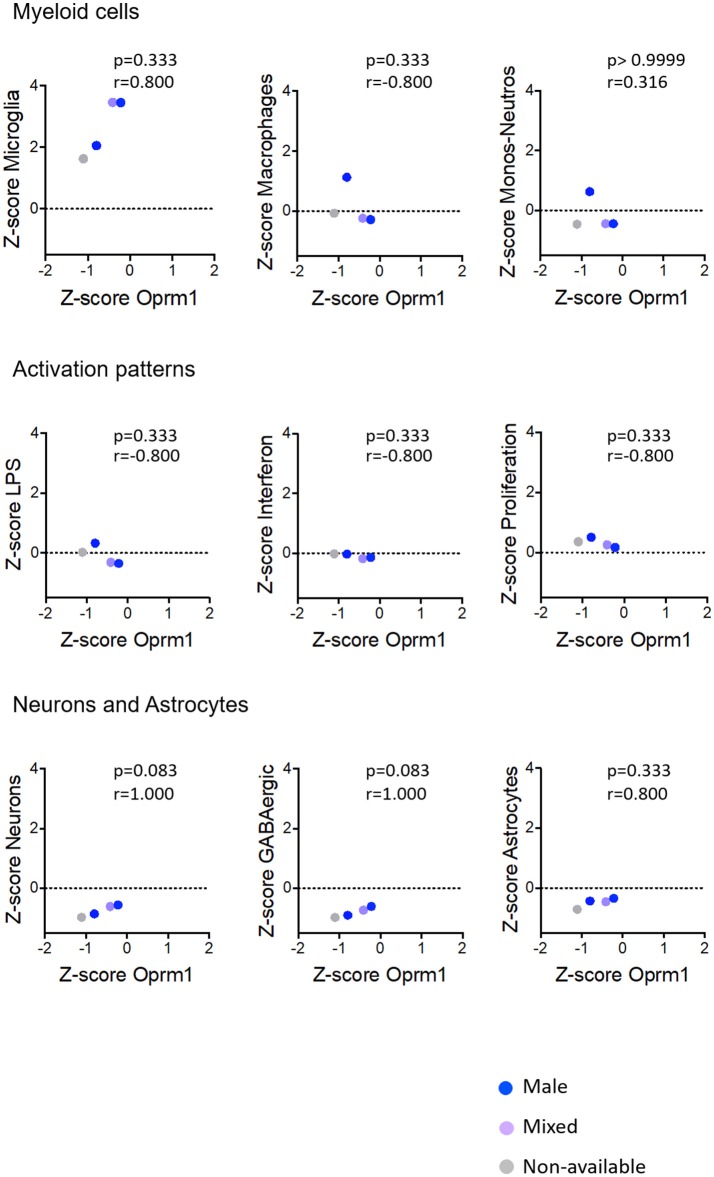
Correlation of Oprm1 gene expression within the Myeloid cells, Activation patterns and Neurons and Astrocytes gene clusters in mouse hippocampus datasets. There is no correlation between Oprm1 expression and all the gene clusters investigated in the mouse hippocampus datasets (*p* > 0.05, ns). Although Oprm1 expression seems to have a strongly positive correlation with the Neurons and Astrocytes gene clusters (*r* = 1 and *r* = 0.800, respectively) this is not statistically significant (*p* > 0.05, ns). All correlations were determined with the Spearman's correlation test.

**Figure 8:**
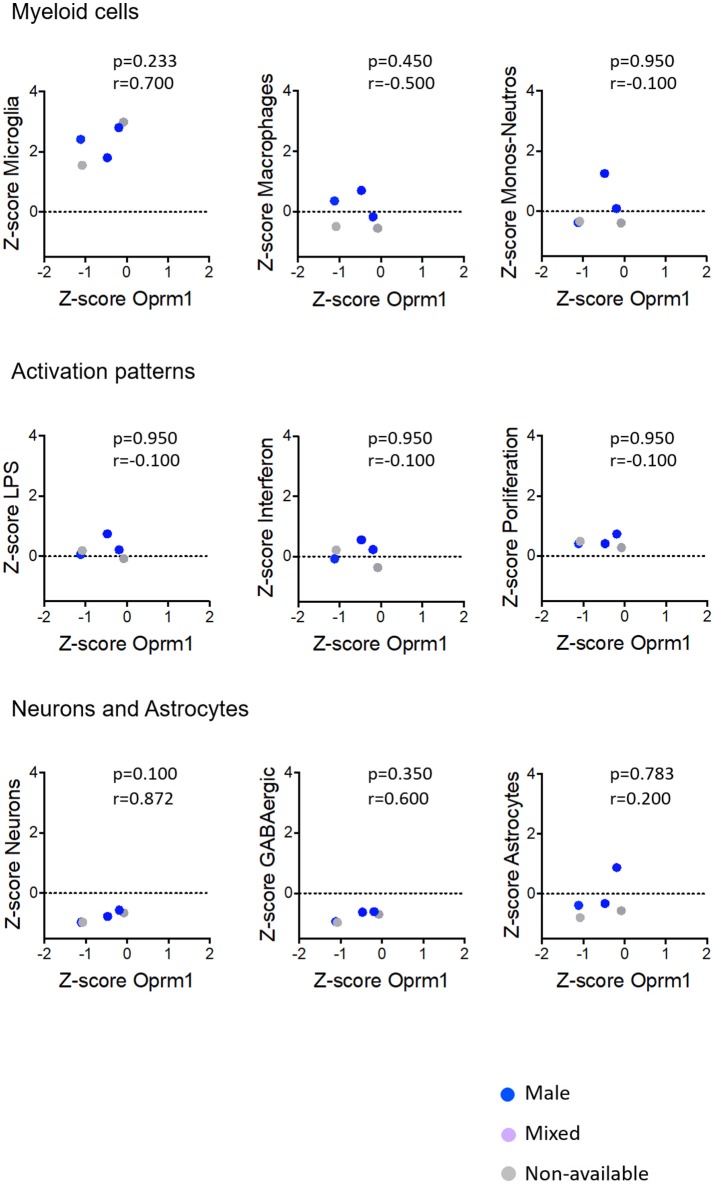
Correlation of Oprm1 gene expression within the Myeloid cells, Activation patterns, and Neurons and Astrocytes gene clusters in rodent spinal cord datasets. Oprm1 expression has a positive correlation (*r* = 0.700) with the Microglia-related genes but which is not statistically significant (*p* > 0.05, ns). Oprm1 expression is negatively correlated (*r* < 0) with Macrophages, Monocytes-Neutrophils- related genes, and Activation patterns gene clusters, but this is not statistically significant (*r* < 0*; p* > 0.05, ns). Oprm1 expression has a positive correlation with the Neurons (*r* > 0.5) which is not statistically significant (*p* > 0.05, ns). There is no correlation between Oprm1 expression and Astrocytes gene clusters (*r* = 0.2*; p* = 0.783, ns). All correlations were determined with the Spearman's correlation test.

**Figure 9:**
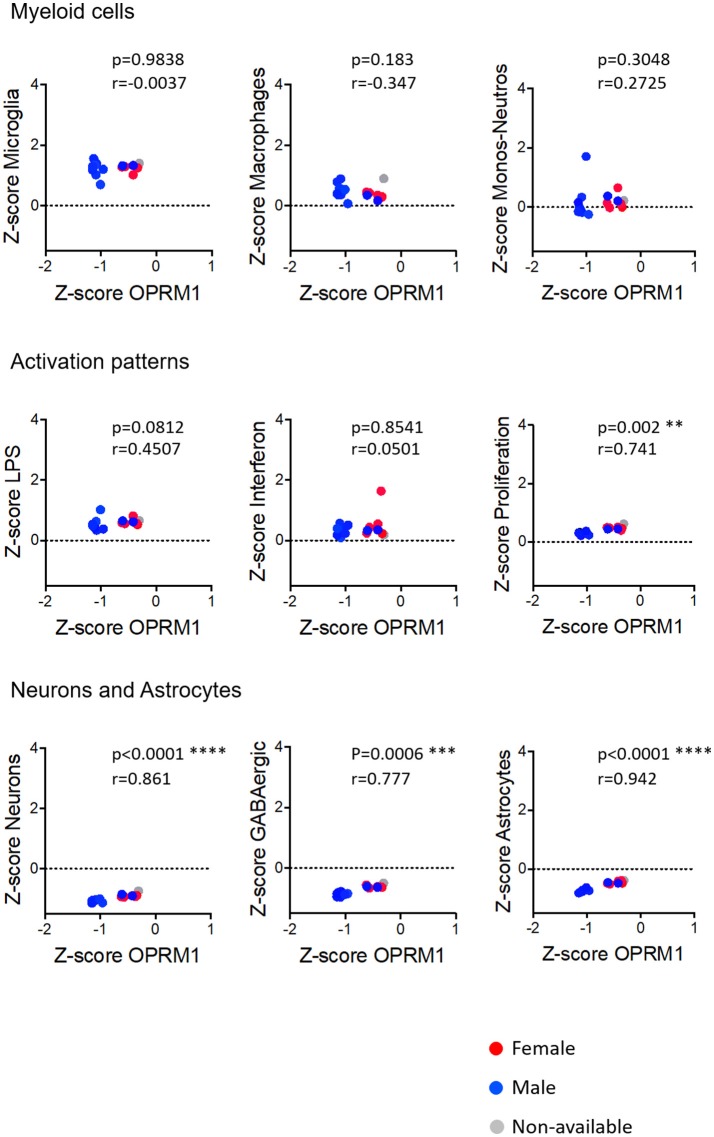
Correlation of Oprm1 gene expression within the Myeloid cells, Activation patterns and Neurons and Astrocytes gene clusters in human cortex datasets. Oprm1 expression had a negative correlation with Microglia- and Macrophage-related genes (*r* < 0) and a positive correlation with the Monocytes-Neutrophils-related genes which were not statistically significant (*p* > 0.05; ns). Within the Activation patterns-related genes, Oprm1 expression has a weak positive correlation (*r* < 0.5) which is not statistically significant for the LPS-related genes (*p* > 0.5). There is a statistically significant positive correlation between Oprm1 expression and the Proliferation-related genes (*r* = 0.741*;* **p = 0.002). There is a statistically significant positive correlation between Oprm1 expression and the GABAergic- (*r* > 0.5*;* ***p = 0.0006), Neurons-, and Astrocytes-related genes (*r* > 0.5*;* ****p < 0.0001).

We investigated the relationships between Oprm1 expression and the expression of myeloid molecular signatures (modules) that are genes found to be co-expressed in the study by Friedman et al. (40). Microglia, in comparison with Macrophages cluster, had inverse trends regarding their correlations with *Oprm1* in the rodent analyses (Figures 5–**9**). The Microglia module was positively correlated with *Oprm1* (mouse cortex, mouse hippocampus, and rodent spinal cord), while the Macrophage cluster was negatively correlated with *Oprm1*.

In whole brain analysis, *Oprm1* expression had a negative correlation with the Macrophage and Monocyte-neutrophil clusters, which was statistically significant with the Macrophage gene cluster only (*p* = 0.030). In the mouse cortex, there was a strong positive correlation between *Oprm1* and the Microglia gene set which was statistically significant only for this cluster (r = 0.955; *p* = 0.0032) but not for the other myeloid sets (Figure 6). No significant correlation was found between the Monocyte-neutrophil gene cluster and any dataset (Figures 5–8).

#### Activation Patterns

We investigated the relationships between the expression of *Oprm1* and of the myeloid activation modules as described (40). There was a negative correlation between *Oprm1* and the LPS gene cluster in the mouse whole brain datasets (Figure 5) and a positive correlation between *OPRM1* and the Proliferation-related gene level for the human cortex datatsets (Figure 9). No correlation was found between *Oprm1* level and the Interferon cluster in any of the dataset studied (Figures 5–9).

#### Neurons and Astrocytes

The analysis for relationships between *Oprm1* expression and expression of the Neuron and GABAergic neuronal signatures indicated no correlation between *Oprm1* and these modules for whole mouse brain, cortex, hippocampus, and rodent spinal cord datasets (Figures 5–8). The *p* and r values obtained from the analysis of hippocampus and spinal cord data suggest that more datasets are required to increase the power of the correlation analysis. For the human cortex datasets, a positive correlation was found between *Oprm1* expression and expression of the Neuronal and Astrocyte gene modules (Figure 9).

### MOR Expression in CX3cr1-eGFP Microglia

#### MOR Expression in Brain and Spinal Cord

**Figure 10:**
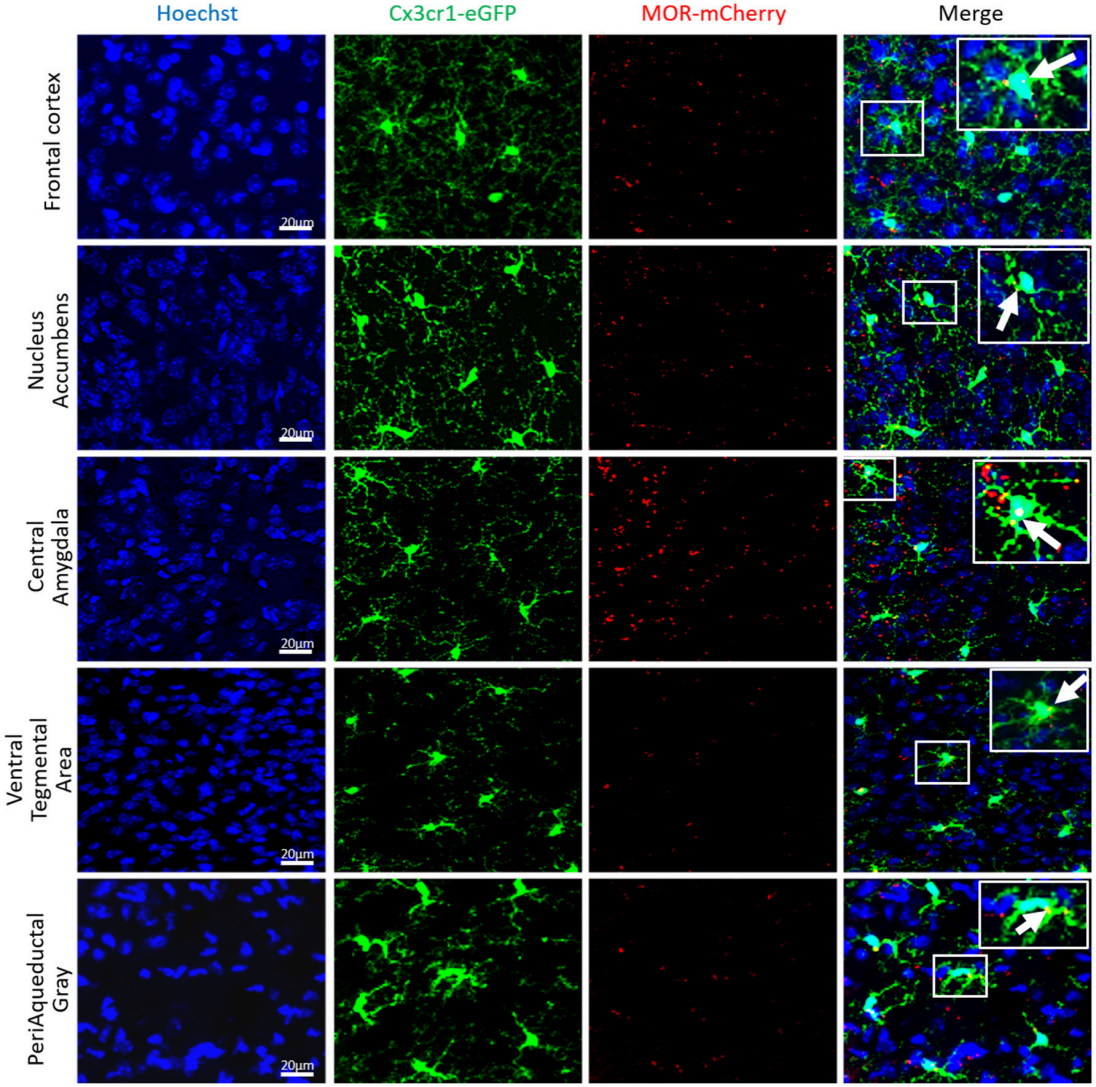
MOR is expressed in mouse brain microglia. Photomicrographs from coronal sections of Frontal cortex (FCx), Nucleus Accumbens (NAcc), Central Amygdala (CeA), Ventral Tegmental Area (VTA), and Periaqueductal Gray (PAG) of Cx3Cr1-eGFP-MOR-mCherry mice show the colocalization (orange) of CX3CR1-eGFP (green), and MOR-mCherry (red) denoted by white arrows. Scale bar = 20 μm.

**Figure 11:**
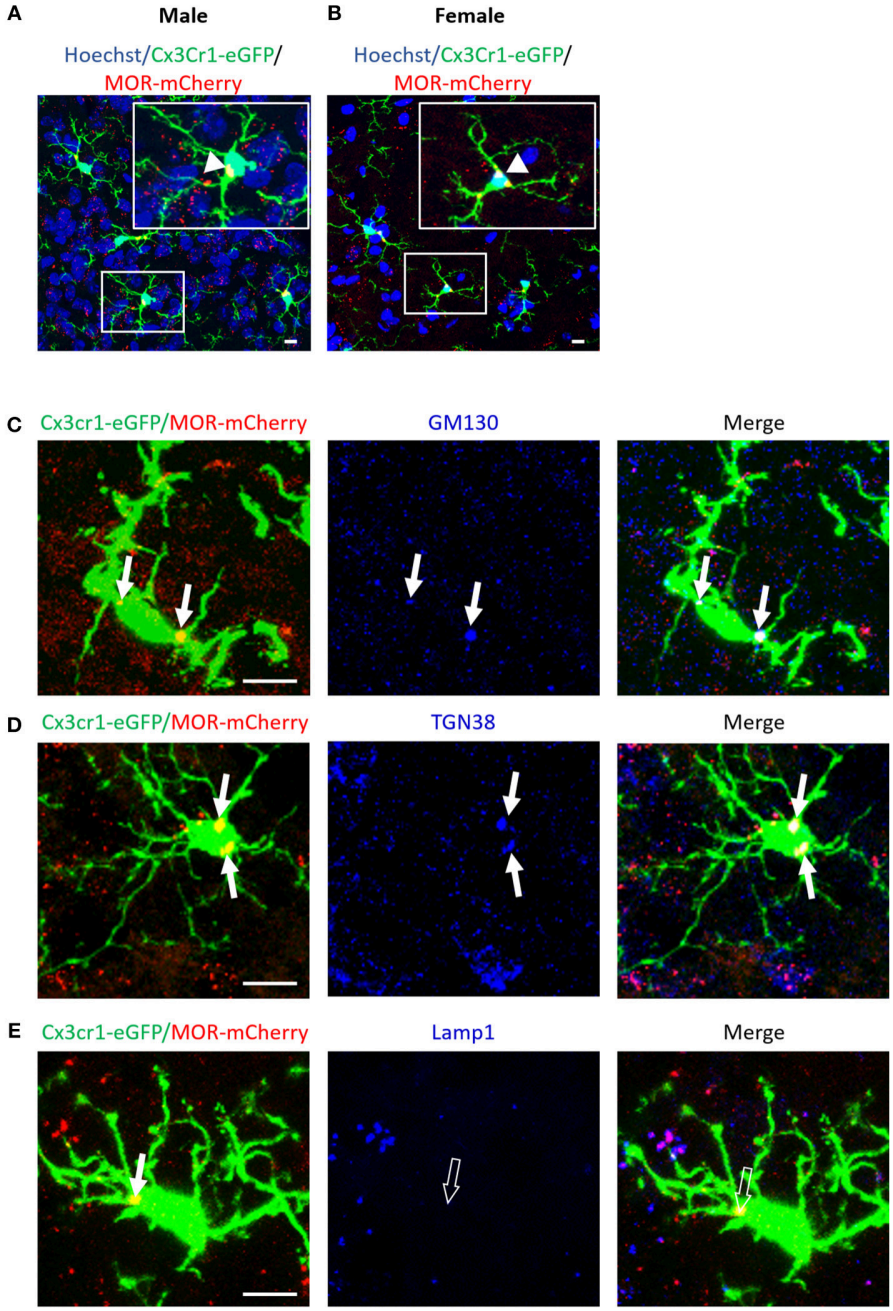
MOR is expressed in microglia of the mouse spinal cord. **(A,B)** Photomicrographs from transverse sections of the lumbar spinal cord, at the level of the dorsal horn, demonstrate a colocalization (orange) of MOR-mCherry (red) with CX3CR1-eGFP-positive microglia (green) in adult male **(A)** and female **(B)** Cx3Cr1-eGFP-MOR-mCherry mice, indicated by white arrow heads. Scale bar = 10 μm. **(C–E)** High magnification photomicrographs of CX3CR1-eGFP positive microglia (green) that colocalize (orange) with MOR-mCherry (red) are indicated with white arrows. Scale bar = 10 μm. **(C)** Anti-GM130 labeling (blue) colocalizes (pink-white) with MOR-mCherry in the cis-Golgi of CX3CR1-eGFP positive microglia, indicated with white arrows. **(D)** Anti-TGN38 labeling (blue) colocalizes (pink-white) with MOR-mCherry in the trans-Golgi of CX3CR1-eGFP positive microglia, indicated with white arrows. **(E)** Anti-Lamp1 labeling (blue) of lysosomes does not colocalize with MOR-mCherry in CX3CR1-eGFP positive microglia, indicated with open arrows. The white (closed) arrow indicate colocalization of CX3CR1-eGFP with MOR-mCherry (orange) alone.

The Figures 10, 11 show that Cx3cr1-eGFP-positive cells display the typical microglia morphology with no neuronal, astrocytic or lymphocytic shapes, confirming previous findings on colocalization of CX3CR1-eGFP with the CD11b or Iba-1 microglia and macrophage markers (37, 74, 75) but not neuronal, astrocytic, or oligodendrocytic markers (76, 77) in the brain. MOR is expressed in microglia of different brain regions in female and male Cx3cr1-eGFP-MOR-mCherry mice, in the Frontal Cortex (FCx), Nucleus Accumbens (NAcc), Central Amygdala (CeA), Ventral Tegmental Area (VTA), and Periaqueductal Gray (PAG) (Figure 10). The percent of microglia expressing MOR-mCherry protein ranges from 35.4 ± 4.1 to 51.6 ± 3.5 (Supplementary Table 5). The percentage of MOR-positive microglia was calculated in the FCx, NAcc, CeA, and PAG of female and male brains. The VTA showed a lower percentage of microglia containing MOR in females than in males which was statistically significant (*p* = 0.0037) (Supplementary Table 5).

In spinal cord dorsal horn, MOR is expressed in microglia of both female and male Cx3cr1-eGFP-MOR-mCherry mice (Figures 11A,B, Supplementary Video 1, Supplementary Table 5). The percentage of microglia expressing MOR ranged from 37.1 ± 2.7 to 44.5 42.4 ± 2.6 in cervical, thoracic and lumbar segments, with no statistical difference between the segments (Supplementary Table 5). There were no statistically significant differences in the percentages of MOR-expressing microglia between males and females with 36.9 ± 2.3% and 36 ± 2.4%, 38.8 ± 2.1% and 42.4 ± 2.5%, and 37.1 ± 2.7% and 39.5 ± 2.3% in cervical, thoracic, and lumbar spinal segments, respectively (Supplementary Table 5).

#### MOR-Golgi-lysosome Localization

To specifically localize MOR within the intracellular compartments of spinal microglia, we labeled Cis-Golgi, trans-Golgi, and lysosomes with GM130, TGN38, and Lamp1 antibodies, respectively. Microglial MOR co-localized with GM-130 (Figure 11C) and TGN38 (Figure 11D) but not with Lamp1 (Figure 11E). Together, this indicates that microglial MOR is localized in cis and trans-Golgi rather than lysosomes.

## Discussion

Altogether the transcriptomics and imaging data show MOR expression in microglia of all analyzed mammalian brain areas and the spinal cord. The expression of Oprm1 transcripts and MOR protein in microglia was shown previously by using RT-qPCR or antibody based techniques, respectively (9–14, 16). Although two papers found no expression of MOR in microglia by using the same above-mentioned techniques (21, 22), a link between microglial activation and MOR expression and function was demonstrated, mostly in *in vitro* studies on murine primary microglia cultures. Low concentrations of morphine and DAMGO (a selective MOR agonist) activated rat microglia and this activation was blocked by the MOR-selective antagonist D-Phe-Cys-Tyr-D-Trp-Arg-Thr-Pen-Thr-NH2 (CTAP) (15). Morphine increased Toll-like Receptors (TLRs) expression which was attenuated by the MOR-selective antagonist D-Phe-Cys-Tyr-D-Trp-Orn-Thr-Pen-Thr-NH2 (CTOP). Additionally, this morphine effect was abolished in cultured microglia of MOR knockout (KO) mice (78). In murine microglia, morphine increased the expression of cytokines and activated the PKCe-Akt-ERK1/2 kinase pathway and these effects were inhibited by *Oprm1* RNA silencing as well as by CTAP (14, 16). A low dose of morphine enhanced NF-kB activity via MOR that was blocked by CTAP while a morphine high dose triggered a TLR4-mediated non-opioid response (18). One study has shown that, *in vivo*, the morphine-induced increase in microglial P2X4R ATP-receptor was blocked by (-)-naloxone but not by its (+)-naloxone enantiomer, implicating microglial MOR rather than TLR4 in OIH (17). MOR implication in OIH has been confirmed at the genetic level with the use of global MOR KO mice (21, 79). Regarding the implication of TLR4 in OIH, the study of TLR4 KO mice has led to controversial results as two papers showed that OIH was abolished in TLR4 KO mice while two others showed that it was maintained (6, 7). Moreover, TLR4-independent activation of NF-kB by morphine was shown together with increased Tumor Necrosis Factor alpha (TNFα), suggesting TNFα signaling as a novel pathway for morphine action on microglia (80). However, whether this effect of morphine on microglia activation occurs directly through the microglial MOR or indirectly via a neuronal MOR still remains to be determined and could be addressed in upcoming studies such as with conditional MOR-KO targeted at microglia or other cell types.

The detection of *Oprm1/OPRM1* in 27 out of 30 rodent datasets and 16 out of 17 human whole genome transcriptomics datasets issued from microglia *ex vivo* validates the presence of MOR transcripts in microglia. From this analysis, we could conclude that in the mammalian central nervous system, microglia of the whole brain, cerebral cortex, hippocampus, and spinal cord all contain *Oprm1/OPRM1* messenger. Single-cell RNA-seq currently allows detection of only a few thousand of the most highly expressed transcripts within a specimen. Therefore, this approach is used for determining differential gene expression among the highly expressed genes and cannot analyze complete transcriptomes (81). As *Oprm1* is overall expressed around or below the median within the reported transcriptomes, the transcripts would not be contained among the few thousands of highly expressed genes detected by single-cell RNA-seq studies. This lack of expression is found also in the Mars-seq datasets (58).

The analysis of the relationships between expression of *Oprm1* and of the different gene clusters led to interesting insights. First, Figures 5–9 show that the z-scores for Microglia-related gene expression are globally above 1 while z-scores for neurons or astrocytes were below 0, indicating a significant enrichment in microglia compared to other cell types. The positive correlation observed between *Oprm1* and the Microglia module in mouse cortex further supports the presence of *Oprm1* expression in microglia. Such a correlation was not found for the mouse whole brain or spinal cord datasets, possibly reflecting the diverse cellular heterogeneity in these tissues compared to a defined region like the cortex. Indeed, the whole brain comprises many distinct regions, and microglia from other brain regions implicated in opioid response or pain, such as VTA, NAcc, or PAG, should be further profiled at the molecular level in order to characterize the global expression of microglial *Oprm1*. Neurons, GABAergic neurons and Astrocytes gene clusters correlate with *OPRM1* in the human cortex datasets but not in datasets from rodents. A significantly higher content of neuronal and astrocyte gene transcripts in FACS-isolated microglia compared to cultured microglia has already been reported, and was suggested to result from synapse-related mRNAs that were phagocytosed by the microglia (19). The same phenomenon may contribute to the positive correlation found between *OPRM1* and Neurons and Astrocytes modules in the human datasets, despite their very low expression levels in the datasets. In addition to the transcriptomic analysis, imaging of the Cx3cr1-eGFP-MOR-mCherry reporter line confirmed MOR expression by microglia of the FCx, NAcc, CeA, VTA, PAG, and spinal cord. Furthermore, co-localization of microglial MOR with cis- and trans-Golgi compartments but not lysosomes strongly supports the protein synthesis and post-translational processing of MOR within microglial cells.

Importantly, as discussed by Richard M Ransohoff, microglia polarization into M1 and M2 subtypes appears too simplistic and does not reflect the diversity of microglia phenotypes or profiles induced by internal and external environmental factors that include central nervous system region, sex, age, and health state revealed by high-throughput analyses (82). Some reports on microglia transcriptomics have defined co-regulated gene subsets or modules to allow for classification of microglia into subtypes expressing high levels of specific gene modules. We analyzed the 47 microglia datasets and established three main activation module profiles related to Interferon, LPS and Proliferation, in relation to *Oprm1* expression. We found a lack of, or a poor correlation between *Orpm1* and these modules. There is a negative correlation between *Oprm1* and the LPS-related module in whole mouse brain datasets, and a positive correlation between *OPRM1* and the Proliferation-related module in human cortex datasets. These low correlations may be explained by the use of datasets from naive, non-stimulated rodents microglia in which the LPS-related, Interferon-related, and Proliferation related genes are expressed at the basal low level.

Several lines of evidence support a functional impact of MOR on microglia activity. Morphine increased spinal microglial p38 and extracellular receptor kinase (ERK) phosphorylation, leading to an augmented microglial production of proinflammatory cytokines, and other pronociceptive molecules as well as their receptors and hence to increased pain (15). Morphine also enhances the activity of microglial ATP-gated P2X7R and P2X4R receptors in the spinal cord, leading to increased Brain-derived neurotrophic factor (BDNF) release from these cells. This in turn downregulates K-Cl co-transporter KCC2 expression in GABAergic neurons enhancing their excitability and thus increased pain (8, 17, 20, 83). Other spinal microglial-mediated responses for morphine analgesic tolerance and hyperalgesia involve large conductance Ca2+-activated K+ channels (84) and genes of the DPA12/TREM2 and potassium intermediate/small conductance calcium activated channels KCNN4 pathways (62). Furthermore, bidirectional cross-talks between chemokine receptors and opioid receptors mediate opioid analgesic tolerance (85). Also, microglial pannexin-1 channels attenuate morphine withdrawal in rodents but are not involved in opioid analgesic tolerance, thus identifying pannexin-1 as a novel mediator of specific morphine actions in microglia (86, 87). Peripheral nerve injury leads to microglial activation in several brain structures involved in pain sensation or emotion including the VTA and amygdala (88). Within the VTA, chronic pain, chronic opiate treatment, and opioid withdrawal induce a dysregulation of BDNF in microglia that impacts on GABAergic neurons and disrupts the dopaminergic pathway leading to defects in reward (89, 90). Therefore, specific regulations in microglia appear to mediate opioid reward and link chronic pain and opioid dependence (91, 92) that may be mediated by MOR activity within different subsets of microglia.

With the use of the Cx3cr1-eGFP-MOR-mCherry reporter mouse, we have shown that, overall, 35–51% microglia express MOR, suggesting that the receptor may be expressed by a particular microglial subset and that only MOR-expressing microglia will respond to opioid treatment. Future studies are necessary to address how MOR expression within different microglial subsets may impact on their functionality, under physiological, and pathological conditions. The specific ablation of the Oprm1 gene in the different subtypes of resting or activated microglia by targeted Cre/Lox technology would require the identification of unique and very selective gene markers for each of these subtypes and the generation of the corresponding Cre mouse lines. In the same line, optogenetic approaches allow the evaluation of opioid signaling and behavior elicited by specific spatiotemporal patterns of MOR activation in neurons (93) that could be applied to microglia. Another aspect of MOR activation is ligand-biased signaling where different ligands stimulate differential cell responses (94, 95). We report an intracellular localization of MOR within the Golgi apparatus of microglia that suggests a local production of MOR in these cells. MOR function in microglia could be investigated by novel biosensors that have been assayed on transfected cell lines and primary neuronal cultures, revealing a cell localization bias for opioid receptor activation by endogenous, and exogenous opioids (96).

As sex differences are an important factor for microglial contribution to pain (35, 36), we investigated MOR expression in microglia from adult females and males. The transcriptomic datasets did not contain enough numbers per sex for a full determination of sex effect, see Figures 5–9. The analysis of brain sections from the Cx3cr1-eGFP-MOR-mCherry mice indicated a comparable ratio of microglia containing MOR in female and male animals in different brain and spinal regions, except in the VTA where significantly lower percentages of microglia expressed MOR in females than males. It would therefore be interesting to determine whether microglial MOR would influence the regulatory mechanisms in the dopaminergic system in females as has been previously described for males in the VTA (89, 90). Also, sex differences in microglia activity were found in the PAG where a significantly higher microglial activation profile was found in females compared to males (97). In the spinal cord, the microglial P2X4R-mediated hyperalgesia showed sexual dimorphism (98). Altogether this suggests that transcriptomic profiles of microglia in chronic pain models or following chronic drug administration may help to identify *Oprm1*-linked mechanisms associated with alterations in microglial functions. However, sex differences may exert a weaker influence on morphine-induced analgesic tolerance and hyperalgesia than on other forms of chronic pain like neuropathic pain. Indeed, these morphine adverse effects occurred similarly in male and female mice (79) and no clear impact was shown on OIH (6).

The question of the factors influencing the opioid effects on microglia physiology should be further explored in the future by taking into account the genetic diversity, sex differences, and pathological states (26). The importance of genetic background on opioid tolerance, hyperalgesia (6), and misuse (99) has been well-established in both clinical and preclinical settings. The rodent datasets analyzed in the present study were from mouse of C57BL/6 genetic background except four datasets issued from other strains, genetic background, or species. In the future, the study of *Oprm1* expression may be broadened to a larger number of rodent strains to get a more comprehensive view. In addition, all human microglia datasets were collected from cortical areas and there is a need for studies on microglia from spinal cord and other brain regions involved in pain and addiction. The correlations between rodent and human transcriptomes may be studied as well. Galatro et al. have shown an overlap between the two species, with similar microglia core genes expressed by both human and mouse microglia including the main markers CX3CR1, P2YR12, and Itgam. There were also dissimilarities due to differences in environment and medical condition as mouse microglia were isolated from healthy mice while human donors suffered from pathologies that led to their death, potentially influencing microglial genes regulation, and expression (65).

Neuroinflammatory mechanisms underlie opioid-induced pain sensitization (6, 7) comprising of reciprocal signaling between neurons and neuroinflammatory cells including microglia (100). OIH, together with abuse liability, contribute to the opioid crisis that is epidemic in North America and becoming prevalent in Europe as well (101). Therefore, the elucidation of mechanisms involved in opioid deleterious effects would help to develop new strategies for safer analgesic clinical intervention. Altogether, this work on microglial *Oprm1* expression and previous studies open the way to the exploration of the role of microglial MOR in response to opiates, in particular to analgesic tolerance, OIH, and physical dependence elicited by their chronic use.

## Data Availability Statement

Data are available on request.

## Author Contributions

TM analyzed data and contributed to the writing of the manuscript. EA, DD, NM, and CG-R designed experiments, analyzed data and contributed to the writing of the manuscript. DR performed experiments. DM provided the MOR-mCherry mouse line and contributed to the writing of the manuscript.

### Conflict of Interest Statement

The authors declare that the research was conducted in the absence of any commercial or financial relationships that could be construed as a potential conflict of interest.

## Abbreviations

BDNF: brain-derived neurotrophic factor
CeA: central amygdala
CTAP: D-Phe-Cys-Tyr-D-Trp-Arg-Thr-Pen-Thr-NH2
eGFP: enhanced green fluorescent protein
FCx: frontal cortex
KO: knockout
MA: microarray
Mars-seq: massively parallel Single-cell RNA-seq
MOR: mu opioid receptor
NAcc: nucleus accumbens
OIH: opioid-induced hyperalgesia
PAG: periaqueductal gray
PBS: phosphate buffer baline
PFA: paraformaldehyde
RNA-seq: RNA-sequencing
TLR: toll like receptor
VTA: ventral tegmental area.
