# Supplementary material for: Microglia Express Mu Opioid Receptor: Insights From Transcriptomics and Fluorescent Reporter Mice
Source: Front Psychiatry. 2019 Jan 4;9:726. doi: 10.3389/fpsyt.2018.00726 (PMC6328486; doi:10.3389/fpsyt.2018.00726)
Supplement: Supplementary file 1 [file Data_Sheet_1.PDF]

Microglia Express Mu Opioid Receptor: Insights from Transcriptomics and Fluorescent Reporter Mice

Tando Maduna, Emilie Audouard, Doulaye Dembélé, Nejma Mouzaoui, David Reiss, Dominique Massotte, and Claire Gaveriaux-Ruff*

* **Correspondence:** Claire Gaveriaux-Ruff: gaveriau@igbmc.fr

Supplementary Table 1. Informations on mouse whole brain microglia datasets

First author, year last author	Reference number	Accession	Strain	Sex	Age (months)	Dissocation	Microglia isolation	Genomics Assay
Wang Y, 2015 Colonna M	41	GSE65067	C57BL/6	Mixed, mostly F	8	Trypsin	FACS $CD11b^{+}$;$Cd45^{Int}$	Affymetrix Mouse Gene 1.0 ST Array
Verheijden S, 2015 Baes M	42	GSE66420	Swiss/Webster	Mixed, mostly F	5	Mechanical	FACS CD11b+;CD45Int	Affymetrix Mouse Gene 1.0 ST Array
Poliani PL, 2015 Colonna M	43	GSE66926	C57BL/6	M	≥2	Trypsin	FACS CD11b+;CD45Int	Affymetrix Mouse Gene 1.0 ST Array
Erny D, 2015 Prinz M	44	GSE67858	C57BL/6	F	2	Mechanical	FACS CD11b+;CD45Int	Affymetrix Mouse Gene 2.1 ST Array
Szulzewsky F, 2015 Kettenmann H	45	E-MTAB-2660	C57BL/6	Mixed	2	Papain	MACS $CD11b^{+}$	Affymetrix Mouse Gene 1.0 ST Array
Pyonteck SM, 2013 Joyce JA	46	GSE37475	C57BL/6	Mixed	3	Collagenase III	FACS $CD11b^{+}$	Affymetrix Mouse Genome 430A 2.0 Array
Bruttger J, 2015 Waisman A	47	GSE68376	C57BL/6	Mixed	3	Papain	FACS CD11b+;CD45Int	RNA-Seq Illumina MiSeq
Lavin Y, 2014 Amit I	48	GSE63340	C57BL/6	F	2	Collagenase D	FACS CD11b+;CD45Int	Derivation of MARS-Seq
Bennett M, 2016 Barres B	49	na	C57BL/6J	Mixed	2	Mechanical	immunopanning CD45+	RNA-seq Illumina NextSeq
Gosselin D, 2014 Glass CK	50	GSE62826	C57BL/6J	M	2	Mechanical	FACS CD11b+;CD45Int	RNA-seq Illumina HiSeq 2000
Krasemann S, 2017 Butovsky O	51	GSE101689	C57BL/6	F	2	Mechanical	FACS FCRLS;CD11b+	RNA-seq Illumina HiSeq500
Zhao D, 2017 Lachman HM	52	GSE69607	C57BL/6	F	1.1	Papain	MACS $CD11b^{+}$	RNA-seq Illumina HiSeq 2500

na, non available
