# Supplementary material for: Microglia Express Mu Opioid Receptor: Insights From Transcriptomics and Fluorescent Reporter Mice
Source: Front Psychiatry. 2019 Jan 4;9:726. doi: 10.3389/fpsyt.2018.00726 (PMC6328486; doi:10.3389/fpsyt.2018.00726)
Supplement: Supplementary file 2 [file Data_Sheet_2.PDF]

Microglia Express Mu Opioid Receptor: Insights from Transcriptomics and Fluorescent Reporter Mice

Tando Maduna, Emilie Audouard, Doulaye Dembélé, Nejma Mouzaoui, David Reiss, Dominique Massotte, and Claire Gaveriaux-Ruff*

* **Correspondence:** Claire Gaveriaux-Ruff: gaveriau@igbmc.fr

Supplementary Table 2. Informations on mouse cortex, hippocampus and striatum microglia datasets

First author, year last author	Reference number	Accession	Strain	Sex	Age (months)	Brain region	Dissocation	Microglia isolation	Transcriptomics Assay
Orre M, 2014 Hol EM	53	GSE74615	C57BL/6	Mixed	15-18	Cortex	Papain	FACS CD11b+;CD45+	Agilent 4x44kv2 Mouse Genome Array
Arumugan TV, 2017 Wells CA	54	GSE77986	C57BL/6	Males	3-6	Cortex	Collagenase	FACS CD11b+;CD45int	Affymetrix Mouse 2.0ST Gene Array WT pico
Grabert K, 2016 McColl B	55	GSE62420	C57BL/6	Males	4	Cortex	Collagenase+Dispase	MACS $CD11b^+$	Affymetrix HT MG-430 PM Array Plate
Friedman BA,2018 Hansen D	40	GSE89482	C57BL/6-Cx3cr1-eGFP/+	Mixed	14-15	Cortex	Accutase	FACS CX3CR1-GFP+	Nugen mRNA-Seq
Srinivasan K, 2016 Hansen D	56	GSE75431	C57BL/6	Mixed	7	Cortex	Accutase	FACS $CD11b^+$	Nugen mRNA-Seq
Srinivasan K, 2016 Hansen D	56	GSE75246	C57BL/6	Females	2	Cortex	Accutase	FACS $CD11b^+$	Nugen mRNA-Seq
Zhang Y, 2014 Wu J	57	GSE52564	FVN/Swiss	Mixed	0.5	Cortex	Papain	CD45 immunopanning	RNA-seq Illumina HiSeq 2000
Matcovitch-Natan O, 2016 Amit I	58	GSE79812	C57BL/6J-Cx3cr1-eGFP/+	na	2	Cortex	Dispomix	FACS CD11bint;CD45Int; CX3CR1-GFP+	RNA-seq Illumina NextSeq500 or HiSeq
Grabert K, 2016 McColl B	55	GSE62420	C57BL/6	Males	4	Hippocampus	Collagenase+Dispase	MACS $CD11b^+$	Affymetrix HT MG-430 PM Array Plate
Friedman BA,2018 Hansen D	40	GSE93179	C57BL/6	Mixed	11-12	Hippocampus	Accutase	FACS $CD11b^+$	Nugen mRNA-Seq
Friedman BA,2018 Hansen D	40	GSE93180	C57BL/6	Males	6	Hippocampus	Accutase	FACS $CD11b^+$	Nugen mRNA-Seq
Matcovitch-Natan O, 2016 Amit I	58	GSE79812	C57BL/6J-Cx3cr1-eGFP/+	na	2	Hippocampus	Dispomix	FACS CD11bint;CD45Int; CX3CR1-GFP+	RNA-seq Illumina NextSeq500 or HiSeq
Grabert K, 2016 McColl B	55	GSE62420	C57BL/6	Males	4	Striatum	Collagenase+Dispase	MACS $CD11b^+$	Affymetrix HT MG-430 PM Array Plate

na, not available
