# Supplementary material for: Microglia Express Mu Opioid Receptor: Insights From Transcriptomics and Fluorescent Reporter Mice
Source: Front Psychiatry. 2019 Jan 4;9:726. doi: 10.3389/fpsyt.2018.00726 (PMC6328486; doi:10.3389/fpsyt.2018.00726)
Supplement: Supplementary file 3 [file Data_Sheet_3.PDF]

Microglia Express Mu Opioid Receptor: Insights from Transcriptomics and Fluorescent Reporter Mice

Tando Maduna, Emilie Audouard, Doulaye Dembélé, Nejma Mouzaoui, David Reiss, Dominique Massotte, and Claire Gaveriaux-Ruff*

*** Correspondence:** Claire Gaveriaux-Ruff: gaveriau@igbmc.fr

Supplementary Table 3. Informations on the rodent spinal cord microglia datasets

First author, year last author	Reference number	Accession	Species	Strain	Sex	Age (months)	Dissocation	Microglia isolation	Transcriptomics Assay
Chiu I 2013 Maniatis T	59	GSE43366	Mouse	C57BL/6-SJL	na	2	Mechanical	MACS $CD11b^+$	Ovation RNA-Seq
Denk F, 2016 McMahon S	60	GSE71133	Mouse	C57BL/6	Males	adult	Mechanical	Percoll gradient	RNA-seq Illumina NextSeq2500
Noristani H, 2017 Perrin FE	61	GSE96055	Mouse	C57BL/6J-Cx3cr1-eGFP/+	Males	3	Trypsin-hyaluronidase	FACS CX3CR1-GFP+	RNA-seq Illumina NextSeq2500
Matcovitch-Natan O, 2016 Amit I	58	GSE79812	Mouse	C57BL/6J-Cx3cr1-eGFP/+	na	2	Dispomix	FACS CD11bint; CD45Int; CX3CR1-GFP+	RNA-seq Illumina NextSeq500 or HiSeq
Jokinen V, 2018 Kalso E	62	na	Rat	Sprague-Dawley	Males	2	Trypsin	MACS $CD11b^+$	RNA-seq Illumina NextSeq High SE

na, not available
