# Supplementary material for: Microglia Express Mu Opioid Receptor: Insights From Transcriptomics and Fluorescent Reporter Mice
Source: Front Psychiatry. 2019 Jan 4;9:726. doi: 10.3389/fpsyt.2018.00726 (PMC6328486; doi:10.3389/fpsyt.2018.00726)
Supplement: Supplementary file 4 [file Data_Sheet_4.PDF]

Microglia Express Mu Opioid Receptor: Insights from Transcriptomics and Fluorescent Reporter Mice

Tando Maduna, Emilie Audouard, Doulaye Dembélé, Nejma Mouzaoui, David Reiss, Dominique Massotte, and Claire Gaveriaux-Ruff*

* **Correspondence:** Claire Gaveriaux-Ruff: gaveriau@igbmc.fr

Supplementary Table 4. Informations on human cortex microglia datasets

First author, year last author	Reference number	Accession	Sex	Age (years)	Pathology	Brain region	Dissocation	Microglia isolation	RNA-seq assay
Zhang Y, 2016 Barres B	63	GSE73721	na	45-63	Epilepsy	Temporal cortex grey matter	Papain	immunopanning CD45+	Illumina NextSeq
Gosselin D, 2017 Glass CK	64	na	F	13	S016: Anaplastic ependymoma - WHO grade III	Frontal cortex	Mechanical	FACS CD11b+;CD45low; CD64+; CX3CR1high	Illumina HiSeq4000 or NextSeq
			F	13	S023: Pilocytic astrocytoma with anaplastic features	Frontal cortex	Mechanical	FACS CD11b+;CD45low; CD64+; CX3CR1high	Illumina HiSeq4000 or NextSeq
			F	13	S009: Neurocytic ganglioglioma with severe cortical dysplasia	Temporal cortex	Mechanical	FACS CD11b+;CD45low; CD64+; CX3CR1high	Illumina HiSeq4000 or NextSeq
			F	15	S017: Negative pathology	Temporal cortex	Mechanical	FACS CD11b+;CD45low; CD64+; CX3CR1high	Illumina HiSeq4000 or NextSeq
			F	16	S040: Aneurysm	Temporal cortex	Mechanical	FACS CD11b+;CD45low; CD64+; CX3CR1high	Illumina HiSeq4000 or NextSeq
			M	17	S012: Cortical dysplasia, grade IC, gliosis/scarring	Parietal cortex	Mechanical	FACS CD11b+;CD45low; CD64+; CX3CR1high	Illumina HiSeq4000 or NextSeq
			M	17	S011: Cortical dysplasia, grade IC, gliosis/scarring	Occipital cortex	Mechanical	FACS CD11b+;CD45low; CD64+; CX3CR1high	Illumina HiSeq4000 or NextSeq
			M	16	S037: Dysembryoplastic neuroepithelial tumor, WHO grade 1	Tumor	Mechanical	FACS CD11b+;CD45low; CD64+; CX3CR1high	Illumina HiSeq4000 or NextSeq
Galatro et al, 2017 Eggen B	65	GSE99074	M	67	S14005: Polyneuropathy, Aortic Aneurysm, asystole	Parietal cortex	Mechanical	FACS CD11bhigh;CD45int	Illumina HiSeq 2500
			M	57	S13067: Multi-system atrophy/Euthanesia	Parietal cortex	Mechanical	FACS CD11bhigh;CD45int	Illumina HiSeq 2500
			M	85	S12112: Ischemia, pneumonia	Parietal cortex	Mechanical	FACS CD11bhigh;CD45int	Illumina HiSeq 2500
			M	102	S12067: Ileus	Parietal cortex	Mechanical	FACS CD11bhigh;CD45int	Illumina HiSeq 2500
			M	81	S12048 Bipolar/Euthanesia	Parietal cortex	Mechanical	FACS CD11bhigh;CD45int	Illumina HiSeq 2500
			M	78	SPM9: Pulmonary edema / Chronic ischemic heart disease	Parietal cortex	Mechanical	FACS CD11bhigh;CD45int	Illumina HiSeq 2500
			M	54	SPM22: Acute gastroenterocolitis	Parietal cortex	Mechanical	FACS CD11bhigh;CD45int	Illumina HiSeq 2500
			M	67	SPM33: Hemopericardium / Break dissecting aortic aneurysm	Parietal cortex	Mechanical	FACS CD11bhigh;CD45int	Illumina HiSeq 2500

na, not available
