# Supplementary material for: Microglia Express Mu Opioid Receptor: Insights From Transcriptomics and Fluorescent Reporter Mice
Source: Front Psychiatry. 2019 Jan 4;9:726. doi: 10.3389/fpsyt.2018.00726 (PMC6328486; doi:10.3389/fpsyt.2018.00726)
Supplement: Supplementary file 5 [file Data_Sheet_5.PDF]

Microglia Express Mu Opioid Receptor: Insights from Transcriptomics and Fluorescent Reporter Mice

Tando Maduna, Emilie Audouard, Doulaye Dembélé, Nejma Mouzaoui, David Reiss, Dominique Massotte, and Claire Gaveriaux-Ruff*

* **Correspondence:** Claire Gaveriaux-Ruff: gaveriau@igbmc.fr

Supplementary Table 5. Detailed statistics on the analysis of MOR+ microglia in the Cx3cr1-eGFP-MOR-mCherry mouse line

Region	Sex	Number of fields counted	Number of cells analyzed	Mean % MOR+ cells	SD	SEM	Lower 95% CI of mean	Upper 95% CI of mean	t	Statistical test	Two-tailed p value	Effect size (Cohen's d)
**Brain**												
Frontal Cortex	Male	14	336	44.07	10.22	2.73	38.17	49.98	16.13	Unpaired t-test	0.155	0.4913
	Female	19	324	38.01	13.66	3.13	31.43	44.59	12.13			
Nucleus Accumbens	Male	13	272	38.20	10.31	2.86	31.97	44.43	13.36	Mann Whitney test	0.057	-0.805
	Female	12	172	45.75	7.89	2.28	40.74	50.77	20.09			
Central Amygdala	Male	11	205	51.59	11.53	3,48	43.85	59.34	14.84	Unpaired t-test	0,24	0.6351
	Female	9	139	44.46	13.97	4,66	33.71	55.18	9.54			
Ventral Tegmental Area	Male	9	92	49.68	14.37	4.79	38.63	60.72	10.37	Unpaired t-test	0.037*	1,0738
	Female	9	85	35.37	12.25	4.08	25.95	44.78	8.66			
Periaqueductal Gray	Male	9	105	45.20	8.53	2.84	38.64	51.76	15.89	Unpaired t-test	0.786	-0,1308
	Female	9	90	46.81	15.17	5.06	35.15	58.47	9.26			
**Spinal cord Dorsal Horn**												
Cervical	Male	14	433	36.84	8.82	2.36	31.74	41.93	15.63	Mann Whitney test	0.920	-0.0061
	Female	10	374	36.89	7.36	2.33	31.63	42.16	15.85			
Thoracic	Male	9	225	42.39	7.65	2.55	36.51	48.27	16.63	Unpaired t-test	0.290	0.4651
	Female	14	355	38.80	7.76	2.07	34.32	43.28	18.71			
Lumbar	Male	15	528	39.54	8.99	2.32	34.57	44.52	17.04	Mann Whitney test	0.442	0.2444
	Female	15	670	37.13	10.66	2.75	31.23	43.04	13.49			

CI, confidence interval; SD, standard deviation; SEM, standard error of the mean.
* indicates statistically significant differences between SEMs.
